# Editorial: Synaptic Assembly and Neural Circuit Development

**DOI:** 10.3389/fnsyn.2018.00030

**Published:** 2018-09-18

**Authors:** Chen Zhang, Jaewon Ko

**Affiliations:** ^1^School of Basic Medical Sciences, Capital Medical University, Beijing, China; ^2^PKU-IDG/McGovern Institute for Brain Research, Peking University, Beijing, China; ^3^Department of Brain and Cognitive Sciences, Daegu Gyeongbuk Institute of Science and Technology, Daegu, South Korea

**Keywords:** synapse, neural circuit assembly, synaptic adhesion molecule, recognition, neuron

Neurons transfer and process neural information via specialized junctional structures called synapses. All synapses in the brain operate by identical molecular and cellular principles, but they differ in their specific properties, depending on brain regions and types of neurons to which synapses are connected. Neural circuits are defined as collections of various types of synapses that form networks to perform specific functions. These circuits receive inputs and yield outputs through the operation of various synapse properties. Despite the attention paid to synapses and neural circuits in neuroscience over the past century, less is understood about synaptic assembly and neural circuit development at the molecular level. Recent increases in understanding are due, at least in part, to significant progress in the functional investigation of *trans*-synaptic adhesion molecules. Synaptic adhesion molecules are regarded as playing fundamental and universal roles in the initiation, assembly, refinement, and elimination of synapses and neural circuits. These molecules are thought to mediate physical and chemical recognition by and among neural cells, and to orchestrate various signaling pathways by interacting with other synaptic proteins. Fowler et al. [SynCAMs], Tan et al. [GPI-anchored IGSFs], Terauchi et al. [FGF22], and Lie et al. [SALMs] discussed their recent progress on the designated vertebrate synaptic adhesion molecules, particularly focusing on their functions in promoting synapse formation. Meanwhile, two papers from Victor Cilleros' group (Tomàs et al.) revealed that presynaptic muscarinic acetylcholine autoreceptors, adenosine autoreceptors, and trophic factor receptors have combined actions with intracellular protein kinases during the neuromuscular junction development and synapse elimination.

Won et al. proposed an intriguing competitive mechanism between protein ligands and heparan sulfates, the latter of which critically mediate synaptic specificity, as extensively discussed by Condomitti and de Wit. Um highlighted the putative roles of these synapse organizers in various glial cell types (astrocytes, microglia, and oligodendrocytes) in the context of shaping GABAergic inhibitory synapses and related neural circuits. Encouragingly, their roles have been recently tested in the context of various neural circuits using transgenic animals, suggesting a bridge between molecular and system neurosciences.

In addition to the synaptic cell-adhesion molecules that function at cellular membranes, various intracellular signaling proteins, scaffolds, and cytoskeletal proteins are critical for synapse assembly and neural circuit architecture. In particular, transcription factors have recently emerged as critical players. Gong et al. showed that the transcription factor, Insm1a, contributes to governing motor neuron development. Meanwhile, Oswald et al. employed various functional approaches to reveal that a network driven by the transcription repressor, FOXP2, is involved in brain disorders, and Tian et al. characterized the role of the FMRP1 protein in long-term synaptic plasticity and spatial learning in rats.

The advances in a variety of neuroscience fields, particularly systems and computational neuroscience, have transformed neuroscience, leading to innovative new insights into our understanding on synaptic assembly and neural circuit development. However, our molecular understanding is still incomplete, and more detailed and sophisticated molecular and cellular approaches should be rigorously applied in the coming years.

## Author contributions

All authors listed have made a substantial, direct and intellectual contribution to the work, and approved it for publication.

### Conflict of interest statement

The authors declare that the research was conducted in the absence of any commercial or financial relationships that could be construed as a potential conflict of interest.

